# Work-related stress in Medical and Dentistry programs

**DOI:** 10.47626/1679-4435-2022-996

**Published:** 2024-08-05

**Authors:** Ivonilda de Araújo Mendonça Maia, Evanisa Helena Maio de Brum, Kevan Guilherme Nóbrega Barbosa, Patrícia Gaspar Mello, Catarina Rodrigues Rosa de Oliveira, Mariúcha Vieira Leite de Jesus, Monalisa da Silva Gomes, Beatriz Almeida Bastos, Sônia Maria Soares Ferreira

**Affiliations:** 1 Curso de Medicina, Centro de Estudos Superiores de Maceió (CESMAC), Maceió, AL, Brazil; 2 Mestrado Pesquisa em Saúde, CESMAC, Maceió, AL, Brazil; 3 Instituto Consciência, Cachoeirinha, RS, Brazil; 4 Curso de Psicologia, CESMAC, Maceió, AL, Brazil; 5 Curso de Medicina, CESMAC, Maceió, AL, Brazil

**Keywords:** psychological stress, teaching, health education, estresse psicológico, ensino, educação em saúde

## Abstract

**Introduction:**

Stress occurs more frequently in groups in which the degree of responsibility and decision-making power play notable roles in society, such as professors and health professionals.

**Objectives:**

To measure and understand the stress of professors in the undergraduate course of Medicine and Dentistry of a private educational institution in northeastern Brazil.

**Methods:**

Observational, descriptive and exploratory study with a quantitative approach was conducted between November 2018 and September 2019. A total of 184 professors participated in the study, answering the following instruments: demographic sociodata questionnaire, Stress Symptoms Inventory and the Preliminary Burnout Identification Questionnaire. The Post-Traumatic Stress Symptoms Scale was applied to the 60 professors with stress. The data obtained were submitted to data analysis through Pearson’s chi-square test and logistic regression.

**Results:**

Stress was present in 40.3% of the professors, with a predominance of the resistance phase (85%) and signs indicative of burnout. There was a significant correlation between the presence of stress and the time of traumatic event either with the individual himself or with some relative and/or close friend. There was no correlation between the traumatic events and Post Traumatic Stress Disorder, although a significant correlation was observed between Post Traumatic Stress Disorder and burnout.

**Conclusions:**

The results point to the need to properly identify and manage stress so that the teaching experience is healthy and conducive to the teaching-learning process.

## Abstract (pt)

**Introdução:**

O estresse ocorre de forma mais recorrente em grupos em que o grau de responsabilidade e poder de decisão perpetram notáveis papéis na sociedade, tais como docentes e profissionais de saúde.

**Objetivos:**

Mensurar e compreender o estresse docente dos cursos de graduação de Medicina e Odontologia de uma instituição de ensino privada do Nordeste do Brasil.

**Métodos:**

Tratou-se de um estudo observacional, descritivo, analítico e exploratório, com abordagem quantitativa no período de novembro de 2018 a setembro de 2019. Participaram do estudo 184 docentes, respondendo aos seguintes instrumentos: um questionário sociodemográfico, o Inventário de Sintomas de Estresse e o Questionário Preliminar de Identificação de *Burnout*. Já a Escala de Sintomas de Estresse Pós-Traumático foi aplicada nos 60 docentes com estresse. Foi realizada a análise de dados através do teste de qui-quadrado de Pearson e de regressão logística.

**Resultados:**

O estresse estava presente em 40,3% dos docentes, com predomínio da fase de resistência (85%) e sinais indicativos de *burnout*. Houve correlação significativa entre estresse e tempo de acontecimento traumático, seja com o próprio indivíduo ou com algum familiar e/ou amigo próximo. Não houve correlação entre o acontecimento traumático e o transtorno de estresse pós-traumático, embora tenha sido observada correlação significativa de transtorno de estresse pós-traumático e *burnout*.

**Conclusões:**

Os resultados apontam para a necessidade de identificar e manejar adequadamente o estresse para que a experiência docente seja salutar e propícia ao processo de ensino-aprendizagem.

## INTRODUCTION

The concept of stress has changed over the last few decades, when Hans Selye described it as a physiological defensive reaction of the organism in response to any stimulus in 1936. Stress, according to Selye, is classified into 4 different stages: alert reaction, resistance reaction, near exhaustion, and exhaustion.^1^

Hence, stress is a psychophysiological process in the body, and the stress reaction is the response of the body to any situation that threatens its homeostasis. This produces the need for modification to overcome the stressor causing the biopsychosocial imbalance.^2^

In this sense, depending on the degree and continuation of the response of the body to possible stressors, stress ceases to be a beneficial mechanism and becomes pathogenic, causing countless harm to the individual. In this way, it can be the precursor to various morbidities, many of them systemic. In addition, chronic stress predisposes to different types of diseases, from headaches to diabetes, hypertension, gastritis, immunodepression, and coronary heart disease.^3,4^ In addition, it is also correlated with the emergence of mental disorders such as anxiety, depression, and substance use.

Pathogenic stress is more common in groups where the degree of responsibility and decisionmaking power perpetuate notable roles in society. This includes professors and workers who deal directly with the health of patients, such as physicians and odontologists.^5,6^

Occupational stress has been reported to undermine the ability of professors to teach and impede their ability to connect with students. In addition, the demotivation resulting from burnout can ultimately lead students to achieve less satisfactory results, and it is negatively associated with an increase in the dropout rate.^6-8^

Furthermore, unaddressed stress can worsen the condition through and become disorders and syndromes, such as burnout syndrome, which is related to chronic stress in the workplace that has not been successfully managed, and post-traumatic stress disorder (PTSD), due to the occurrence of a traumatic event.^9,10^

The overlap between teaching and health care duties leads to the hypothesis that these workers are subject to situations of greater emotional exhaustion. These exhausting situations may be related to confronting the suffering and death of patients, and difficulties in interpersonal relationships with patients.^11,12^

When health care workers experience chronic stress, negative repercussions on their quality of work, such as patients are less likely to adhere to treatment and complain about poor quality of care are common and well documented in the literature.^11-14^

However, most of the existing studies on stress have focused on health workers in isolation, without correlating the stress that exists in multidisciplinary teams. Likewise, almost no correlation has been found between the effects of stress on health care professors and their professional training profile.^15^

It is therefore essential to measure and understand the stress of professors of Medicine and Dentistry at a higher education institution in Northeastern Brazil, with a view to (1) profiling the sample, (2) measuring and classifying the prevalence and intensity of stress among professors, (3) understanding the triggering, aggravating, or protective factors of teaching stress and (4) correlating teaching stress with burnout syndrome and PTSD.

## METHODS

This is an exploratory, applied study with descriptive and analytical objectives, an *ex post facto* procedure and a quantitative approach conducted between November 2018 and September 2019. Questionnaires were individually answered at teaching meetings. The study included 184 professors, 139 from the Medical faculty and 45 from the Dentistry faculty. Among the professors who signed the informed consent form (ICF) and participated in the questionnaire, 113 were from the Medical faculty and 36 were from the Dentistry faculty.

The sociodemographic data questionnaire included sociodemographic data and the Critério de Classificação Econômica Brasil (Brazil Economic Classification Criterion), according to the Pesquisa de Orçamento Familiar (POF, Household Budget Survey) of the Instituto Brasileiro de Geografia e Estatística (IBGE, Brazilian Institute of Geography and Statistics).

The Lipp’s Stress Symptom Inventory for Adults (LSSI) has been validated in Brazil and assesses stress, indicating which stage the individual is at: alertness, resistance, near exhaustion, or exhaustion. It is divided into three tables which assess symptoms of stress with psychological and/or physical manifestations in the previous 24 hours (15 items); previous week (23 items); and previous month (15 items).^16^

The Questionário Preliminar de Identificação da Burnout (QPIB - Preliminary Burnout Identification Questionnaire) is a tool validated in Brazil which aims to identify burnout through 20 self-reported questions to be answered on a 5-point Likert scale (1: never; 5: daily). The sum of the scores is 21 to 40 points (possible burnout); 41 to 60 (early burnout); 61 to 80 (onset); and 81 to 100 (burnout).^17^ It was applied to 113 professors: those who showed stress on the LSSI (to differentiate stress from burnout) and those who, despite not being diagnosed with stress, reported that they or a family member or close friend had had a traumatic experience.

The Screen for Posttraumatic Stress Symptoms (SPTSS) is a self-report tool adapted and translated into Brazilian Portuguese, with 17 items in which the participant is required to answer how often they have experienced those events in the previous 2 weeks (0 = never; 10 = always). It aims to match each item to the symptoms included in the diagnostic criteria for PTSD proposed in the 4th edition of the Diagnostic and Statistical Manual of Mental Disorders (DSMIV), which the author refers to as: a) revisiting or re-experiencing, b) avoidance/numbness, and c) increased excitability.^18^ This questionnaire was applied to all participants who reported having experienced or who reported having a family member or close friend who had experienced a traumatic event in the previous 3 months and/or the past year (113 professors). The aim of the application was to specify and differentiate cases of individuals with stress from cases of those with PTSD, since the presence of the disorder can be a confounding variable in the results.

The data was analyzed using Pearson’s chisquare and binary logistic regression. This study was conducted in accordance with the Declaration of Helsinki and Resolution No. 466/2012 of the Conselho Nacional de Saúde (Brazilian National Health Council), and was submitted to Plataforma Brasil for approval (No. 02396418.8.0000.0039). Participants were provided with ICFs and codes were used to guarantee their anonymity.

## RESULTS

Among the 139 professors on the Medical faculty, 113 agreed to participate in the study, equivalent to 81.29% of the professors on the program. In Dentistry faculty, 36 professors agreed to participate, representing 80% of the professors. As for the characteristics of the sample, ages ranged from 27 to 63 years, with a mean of 40.28 years and standard deviation ± 8.356 years. Women (59.1%), married (69.1%), mixed race (48.9%), place of birth Maceió, AL, Brazil (63.5%), living in their own home (78.2%), living with their spouse (46.3%), holding a master’s degree (36.9%), and socioeconomic status B1 (34.1%) prevailed (Table 1). In the statistical analyses of the association between sociodemographic variables, an association was found between stress and ethnicity (p = 0.003) and between stress and place of birth (p = 0.048) (Table 1).

**Table 1:** Sociodemographic data of Medical and Dentistry professors and correlation with stress - Pearson’s chi-squared test

Variable	n	Stress				p-value
		Yes	%	No	%	
Age (years)	145	59	40.70	86	59.30	0.066
27 to 38	77	32	41.60	45	58.40	0.227
39 to 63	68	27	39.70	41	60.30	0.165
Sex	149	60	40.30	89	59.70	0.052
Male	61	20	32.80	41	67.20	0.027
Female	88	40	45.50	48	54.50	0.487
Place of birth	137	54	39.40	83	60.60	0.043
Alagoas	91	34	37.40	57	62.60	0.048
Other state	46	20	43.50	26	56.50	0.471
Ethnicity	141	55	39.00	86	61.00	0.033
White	63	32	50.80	31	49.20	0.918
Non white	78	23	29.50	55	70.50	0.003
Marital status	149	60	40.30	89	59.70	0.052
Unpartnered	40	17	42.50	23	57.50	0.440
Partnered	109	43	39.40	66	60.60	0.072
Residence	145	59	40.70	86	59.30	0.066
Owned	115	50	43.50	65	56.50	0.253
Rented	30	9	30.00	21	70.00	0.072
Place of birth	148	59	39.90	89	60.10	0.043
Maceió	94	36	38.30	58	61.70	0.063
Inland Alagoas	17	6	35.30	11	64.70	0.325
Other state	37	17	45.90	20	54.10	0.688
Lives	147	59	40.10	88	59.90	0.051
on their own	25	12	48.00	13	52.00	0.871
with parents	7	3	42.86	4	5714	0.763
with a spouse	69	26	37.70	43	62.30	0.094
with their children	7	2	28.57	5	71.43	0.361
with relatives	53	21	39.60	32	60.40	0.217
in an institution	0	0	0.00	0	0.00	
Otherwise	2	1	50.00	1	50.00	1.000
Educational background	149	60	40.27	89	59.73	0.052
Specialization	44	17	38.64	27	61.36	0.218
Master’s degree	55	25	45.45	30	54.55	0.583
Doctorate	36	14	38.89	22	61.11	0.277
Post-doctorate	14	4	28.57	10	71.43	0.191
Social class	147	58	39.45	89	60.54	0.036
A	39	13	33.33	26	66.67	0.088
B1	48	18	37.50	30	62.50	0.157
B2	47	21	44.68	26	55.32	0.552
C1	7	4	5714	3	42.86	0.763
C2	5	2	40.00	3	60.00	0.723
D-E	1	0	0.00	1	100.00	0.479

The minimum length of time teaching was 0.5 years, and the maximum was 30 years, with a mean of 8.11 years. On the Medical faculty, the maximum teaching time was 30 years, and on the Dentistry faculty it was 20 years. In both cases, the minimum teaching time was 0.5 years. We found that 76.4% of the professors also worked as a health care worker, with lengths of service from 1 to 37 years (mean 15.43 years). The maximum length of service was 35 years on the Medical faculty and 20 years on the Dentistry faculty (Table 2).

**Table 2:** Professional data of Medical and Dentistry professors and presence/absence of stress

Variable	n	Stress				p-value
		Yes	%	No	%	
Do you work as health care worker?	148	60	40.50	88	59.50	0.060
Yes	113	42	37.20	71	62.80	0.026
Yes	35	18	51.40	17	48.60	0.890
Length of service as a professor (years)	143	59	41.30	84	58.70	0.087
< 1 year	1	1	100.00	0	0.00	0.479
1 to <5	61	27	44.26	34	55.74	0.465
5to<10	32	15	48.87	17	53.12	0.773
10 to <15	19	8	42.10	11	57.89	0.577
15 to < 20	18	5	27.78	13	72.22	0.123
20 to 30	11	3	27.27	8	72.72	0.220
>30	1	0	0.00	1	100.00	0.479
Length of service as a health care worker (years)	120	44	36.70	76	63.30	0.016
1 to 13	63	23	36.50	40	63.50	0.079
14 to 37	57	21	36.80	36	63.20	0.104
Faculty	149	60	40.27	89	59.73	0.052
Medical	113	45	39.82	68	60.17	0.077
Dentistry	36	15	41.70	21	58.30	0415

Among the 113 Medical professors, 45 (39.82%) had stress, and among the 36 Dentistry professors, 15 (41.66%) had stress. As a result, stress was present in 40.3% of all Dentistry and Medical professors.

The stage of resistance (84.44%) predominated among the Medical professors who participated in this study, followed by near-exhaustion (6.6%), alertness (6%), and exhaustion (2.2%). In Dentistry, resistance was also the predominant stage (86.66%), followed by Therefore, combining the data collected from alertness (13.33%). No Dentistry professors were in the Medical and Dentistry professors, 85% of them the near-exhaustion or exhaustion stages. had stress in the resistance stage, followed by the alert stage (8.3%), near exhaustion (5%), and exhaustion (1.7%).

This study carefully screened the 113 Medical and Dentistry professors who had indications of stress on the LSSI and/or reported having experienced trauma (either in their personal life, family life, or a close friend) on the QPIB. The aim was to differentiate between participants with stress and those with burnout syndrome. They were also tested on the SPTSS, to discriminate between participants with PTSD and those with stress.

According to the data obtained, 41.7% of the professors with stress who responded to the QPIB were likely to develop burnout, while signs of burnout were found in 58.4% of the 60 professors with stress who responded to the QPIB (46.7% of whom had burnout in the early stages and 11.7% of whom had burnout).

In relation to the participants in the study being both professors and physicians or dental surgeons, statistical analysis was conducted using Pearson’s chi-squared test. There was a significant correlation between teaching stress and being a health care worker (p = 0.026) (Table 3). It was concluded that 37.20% of the professors had stress, showing that professors are more predisposed to stress because they combine the roles of health care workers with teaching.

**Table 3:** Correlation between professional data and professor stress - Pearson’s chi-square test

Variable	n		Stress			p-value
		Yes	%	No	%	
Do you work as a health care worker?	148	60	40.50	88	59.50	0.06
Yes	113	42	37.20	71	62.80	0.026
No	35	18	51.40	17	48.60	0.89
Length of service as a professor (years)	143	59	41.30	84	58.70	0.087
< 1	1	1	100.00	0	0.00	0.479
1 to < 5	61	27	44.26	34	55.74	0.465
5 to < 10	32	15	48.87	17	53.12	0.773
10 to 14	19	8	42.10	11	57.89	0.577
15 to 19	18	5	27.78	13	72.22	0.123
20 to 30	11	3	27.27	8	72.72	0.22
Over 30	1	0	0.00	1	100.00	0.479
Length of service as a health care worker	120	44	36.70	76	63.30	0.016
1 to 13	63	23	36.50	40	63.50	0.079
14 to 37	57	21	36.80	36	63.20	0.104
Faculty	149	60	40.27	89	59.73	0.052
Medical	113	45	39.82	68	60.17	0.077
Dentistry	36	15	41.70	21	58.30	0.415

In relation to having experienced a traumatic event, 59.06% of the professors reported that they had experienced a traumatic situation. On the other hand, 30.87% of Medical and Dentistry professors reported having experienced a traumatic event involving a family member or close friend. Among those who answered this question affirmatively, 42% reported having experienced at least 1 episode of violence or accident; 36.4% had experienced some form of bodily injury; 12.5% had been threatened with death; and 9.1% had a serious sexual violation. The traumatic event in question occurred within the previous 3 months for 25.6% of the professors who had experienced the traumatic event, and 71.8% had experienced the traumatic event within the previous year. However, 2.6% of the professors who had experienced a traumatic event left this field unanswered (Table 4).

**Table 4:** Post-traumatic stress disorder (PTSD) in Medical and Dentistry professors who reported experiencing a traumatic event

Variable	PTSD				n^*^	p-value
	Yes	%	No	%		
Positive exposure to trauma	3	8.10	34	91.90	37	< 0.0001
In the previous 3 months	1	9.10	10	90.90	11	0.023
In the previous year	2	7.70	24	92.30	26	0.0003
Trauma in the family or with a close friend	3	11.50	23	88.50	26	0.001
In the previous 3 months	0	0.00	6	100.00	6	0.039
In the previous year	3	15.00	17	85.00	20	0.009
Perceived stress	7	11.66	53	88.33	60	< 0.0001
Burnout	9	14.51	53	85.48	62	< 0.0001
No signs of burnout	0	0.00	1	100.00	1	0.479
May experience burnout	1	2.08	47	97.91	48	< 0.0001
Early burnout	2	7.41	25	92.59	27	0.0002
Burnout onset	4	5714	3	42.85	7	0.763
Burnout developed	1	0.00	0	100.00	1	0.479

* Professors with no report of a traumatic event and professors with no stress were excluded.

When exploring the characterization of the traumatic event with the participants (Table 4), the perception of the occurrence of the traumatic event, which had triggered stress, was investigated in terms of its relationship with work, and for 34.21% of the interviewees with stress, the traumatic event was related to work, while for 36.90% of the professors with stress, the traumatic event influenced their work. As for whether the traumatic event had happened to a family member or close friend, 41.67% of the total number of stressed professors answered affirmatively. The period in which the traumatic event with a family member or close friend occurred was in the previous 3 months for 23.53% and the previous year for 76.47% of those who answered positively to this question.

As for the type of traumatic event, 42.04% of the professors reported experiencing violence or an accident, 12.5% death threats, 36.37% bodily injury, and 9.09% sexual violation. Among the 11 professors who reported having experienced a death threat, 54.54% showed stress on the LSSI, and 4 professors who reported having experienced sexual violation showed stress (50%). However, no significant correlation was found between stress and the type of traumatic event. Among professors who had experienced a traumatic event with a family member and/or close friend, 47.82% reported having experienced violence and/or an accident, 26.09% a death threat, 19.56% a bodily injury, and 6.5% a serious sexual violation, and for 26.31% the event had occurred in the previous 3 months; of these, 70% had LSSI scores consistent with stress. Even so, no significant correlation was found (Table 3).

Among the 12 participants who reported having a family member or close friend who had been threatened with death, 75% had LSSI scores consistent with stress. Another 3 participants reported having a family member or close friend who had been seriously sexually assaulted, and 100% of them had LSSI scores consistent with stress.

The SPTSS was applied to 113 participants who reported experiencing a traumatic event and/or showed stress on the LSSI, to differentiate whether they had stress or PTSD. The results showed that no correlation was found with PTSD, since 91.9% of those who had experienced a traumatic event did not have PTSD, and 85% of those who had a family member or close friend who had experienced a traumatic event also did not have PTSD, as shown in Table 4.

The level of burnout was found to have a significant correlation with PTSD (p < 0.01), and 57.14% of the professors who had PTSD on the SPTSS showed signs of initial burnout (Table 4).

The interpretation of the data using binary logistic regression showed that there was a correlation between the variables analyzed when the p-value was < 0.05. The correlation coefficient values ranged from -1, indicating a perfect negative correlation, to +1, indicating a perfect positive correlation (Table 5).

**Table 5:** Estimated Pearson correlation between variables

Variable 1	Variable 2	n	Correlation coefficient	p-value
Length of service as a professor	Length of service as a health care worker	114	0485	< 0.001
Length of service as a professor	Stress	143	0.140	0.095
Length of service as a professor	Burnout	103	-0.017	0.858
Length of service as a professor	Stage of burnout (level)	103	-0.054	0.585
Length of service as a professor	PTSD	103	0.068	0.490
Length of service as a health care worker	Stress	120	0.098	0.287
Length of service as a health care worker	Burnout	88	-0.008	0.939
Length of service as a health care worker	Stage of burnout (level)	88	-0.037	0.731
Length of service as a health care worker	PTSD	86	-0.013	0.908
Stress	Burnout	111	0.054	0.573
Stress	Stage of burnout (level)	111	-0.115	0.231
Stress	PTSD	109	0.107	0.268
Burnout	Stage of burnout (level)	108	-0.880	< 0.001
Burnout	PTSD	108	0.221	0.021
Stage of burnout (level)	PTSD	108	-0.421	< 0.001

PTSD = post-traumatic stress disorder.

Thus, the length of service as a professor and the length of service as a health care worker had a weak to moderate positive correlation (i.e. the longer the length of service as a professor, the longer the length of service as a health care worker). The value obtained on the burnout score and the value obtained on the PTSD score had a weak positive correlation (i.e. the higher the score consistent with signs of burnout, the higher the score suggestive of PTSD). Finally, the value corresponding to the stage of burnout and the value obtained on the PTSD score had a weak to moderate negative correlation (i.e. the higher the score related to the stage of burnout, the lower the score suggestive of PTSD) (Table 6).

**Table 6:** Estimated parameters used in modeling the probability of post-traumatic stress disorder as a function of other conditions obtained from medical professors

Variables	Regression coefficient	Odds ratio (IC95%)	p-value
Stress	-0.287	0.75 (0.127-4.426)	0.752
Burnout	1.400	4.05 (0.167-98.332)	0.389
Stage of burnout	2.728	15.29 (2.513-93.089)	0.003

95%CI = 95% confidence interval.

Among the participants, 41.5% had already had psychological or psychiatric treatment. Among 82.4% of professors who had already had psychological treatment, 2% had already had psychiatric treatment and 15.7% had had both. Based on the data collected, no significant correlation was found between the presence of stress and a history of psychological/ psychiatric treatment. It was found that 99.3% of the professors do leisure activities.

## DISCUSSION

The results show that the mean length of service as a health care worker was 15 years. Thus, it can be inferred that the participants in the study have already reached their professional maturity. It is worthy of note that the length of service has previously been considered a stressor,^19^ since the longer you work in a stressful job, the more stress you experience in the workplace and the greater the wear and tear associated with stress.

However, unlike what has been reported previously,^19^ no significant correlations were found using Pearson’s chi-square test between stress and length of service as a professor or between stress and teaching (for the analysis of each program, only professors from the respective program were considered) (Table 2).

No statistical significance was found between the social class of professors and stress, which can be explained because most professors (93.2%) belong to social classes A and B, have better purchasing power, and more resources. Only 3.4% of professors are in classes D and E.

The increased purchasing power and social status of professors contributes to no significant correlation between social class and stress. This seems to be the case for the sample in this study, since, according to a report by the World Health Organization (WHO) in partnership with the Gulbenkian Global Mental Health Platform,^20^ poverty and a decline in purchasing power, with a consequent decrease in social class, are associated with an increased risk of developing pathogenic stress and common mental disorders.

Among the Medical professors, 39.82% were stressed, as were 41.66% of the Dentistry professors. As a result, 40.3% of all Dentistry and Medical professors had stress. These figures are very similar to those found in a cross-sectional study on stress among professors at a medical school,^21^ where 31% of the Medical professors at that institution reported high overall levels of perceived stress at work.

Similarly, another study^22^ analyzed the levels of stress, anxiety, and burnout among different medical specialties in Brazil and found 44.9% of stress in a sample of 606 participants.

Dentistry professors with stress (41.66%) were also quite similar, only slightly higher than the 32.61% found in a previous study^23^ of Dentistry professors with stress in a study conducted in the United Kingdom.

The predominant stage of stress among the Medical professors participating in this study was resistance (84.44%). This corroborates the findings in the literature. In the resistance stage, the body uses a great deal of energy to try to re-establish internal homeostasis, causing symptoms characteristic of the resistance stage to manifest in the professors.^22,24^ A previous study^22^ using the Depression, Anxiety, and Stress Scale (DASS^21^) also obtained similar data in a Brazilian academic health system in relation to the stage of stress the participants were experiencing.

The predominant stage among Dentistry professors was also resistance (86.66%). Comparing the data in this study with previous data^24^ dental surgeons experience in their academic routine, resistance also prevailed with 85.7% of workers experiencing this stage of stress.

Combining the data obtained from Medical and Dentistry professors, resistance prevailed among 85% of professors with stress (Table 4). The similarity between the data from Dentistry and Medical professors in the current study is because both programs have many stressors in common, given that Dentistry and Medical professors must not only teach theoretical classes, but also tutor and teach, conduct practical activities in the laboratory, and attend seminars and congresses to remain updated, all while practicing clinically. Furthermore, they have the duty to conduct research, do experiments, and write scientific literature, which is why the many stressors are common to both programs.^25^

We can suppose from the fact that 41.7% of the professors were likely to have burnout, and that 58.4% of the 60 professors with stress had signs of burnout, that despite most professors having been considered to be in the resistance stage, their perceived stress is correlated with burnout, indicating that their situation may be more serious than that experienced by the professors.

A significant correlation between stress among professors and working as a health care worker (p = 0.026) (Table 6) shows that professors are more predisposed to stress because they combine their roles as health care workers with teaching. This finding disagrees with the conclusion of a previous study^26^ which reviewed the literature on the subject.

Although many studies have shown high levels of stress in professors, physicians, and dental surgeons, with rates significantly higher than those of the general population, no study could be found showing that physicians and dental surgeons with undergraduate or postgraduate teaching duties had more stress than their peers who did not teach.

However, it has previously been found that the stress of workers who fulfill the dual role of teaching and acting as physicians or dental surgeons can worsen in specific situations.^27^ It has been observed that stress worsens in workers with dual roles when managers add a heavy workload, making professors feel restricted in their functions and autonomy.

The reasons the authors^27^ described for increased stress in this category were the requirement for professors to provide objective reports on their students’ progress; the responsibility of the professor for trainee performance; reduced decision-making autonomy; greater management control over the professor activity; and threats if the rules imposed were not met.

In order to find out whether significant differences existed in stress levels between dental surgeons from different areas, some authors^23^ conducted a one-way analysis of variance (ANOVA), finding that the level of stress reported did indeed vary according to the dental surgeons area of expertise. However, dental surgeons who also worked as professors were no more stressed than other Dentistry professionals, corroborating the findings of this study.

Among the participants, 41.67% reported having experienced a traumatic event with a family member or close friend. In view of this, the risk of developing PTSD and potential aggravating risks should be considered. According to a cross-sectional study with 1,026 doctors,^28^ the presence of PTSD is directly related with burnout syndrome among health care workers, and exposure to multiple life-threatening events was described as the most common traumatic stressor among the physicians studied. The authors concluded that the potential risk factors for PTSD differed, although hospital culture, hospital support, and salary were overlapping risk factors.^28^

Meanwhile, 99.3% of professors were found to engage in leisure activities. According to another study,^29^ certain leisure activities serve as precursors to different types of coping strategies and social support. In particular, engaging in social activities was identified as the strongest predictor of coping strategies and social support, and physical activity was a strong predictor of the avoidance coping strategy.

The authors therefore conclude that participating in social and outdoor activities provides important means for participants to develop the ability to cope with stress and create social support systems.^29^ Despite such significant findings showing the importance of leisure activities as a stress protection factor, no significant correlations were found in this study between the absence of stress and any specific leisure activity.

This study is mainly limited to the cross-sectional nature of the method. Cross-sectional studies do not allow causality to be inferred and should therefore be interpreted with caution. In addition, we used an exclusively quantitative approach, which does not allow us to assess issues related to subjectivity and the perception of professors about occupational stress.

## CONCLUSIONS

This study found that Medical and Dentistry professors experienced stress, predominantly in the resistance stage. Among the sociodemographic factors, stress was correlated with ethnicity. Teachers who reported being stressed had signs of burnout syndrome.

The presence of stress was not associated with the traumatic event that occurred to the individual or to a family member and/or close friend. However, the traumatic event was significantly correlated with PTSD. PTSD was associated with burnout syndrome, showing that PTSD is related to burnout syndrome and vice versa. Thus, high levels of stress among professors point to the need for appropriate stress management so that they can experience teaching as a healthy experience, conducive to the teaching-learning process.

## References

[1] Silva RM, Goulart CT, Guido LA. (2018). Evolução histórica do conceito de
estresse. Revisa (Online).

[2] Bassols AMS. (2014). Estresse, ansiedade, depressão, mecanismos de defesa e coping dos
estudantes no início e no término do curso de medicina na
Universidade Federal do Rio Grande do Sul [Tese de Doutorado].

[3] McCarty R., Fink G (2016). Stress: Concepts, cognition, emotion, and behavior.

[4] Li J, Zhang M, Loerbroks A, Angerer P, Siegrist J. (2015). Work stress and the risk of recurrent coronary heart disease
events: A systematic review and meta-analysis. Int J Occup Med Environ Health.

[5] Santos CLM, Rodrigues CLP, Silva LB, Bakke HA, Leite ASM, Leal MMA. (2011). Fatores de estresse na atividade de médicos em João
Pessoa (PB, Brasil). Prod.

[6] Shen B, McCaughtry N, Martin J, Garn A, Kulik N, Fahlman M. (2015). The relationship between teacher burnout and student
motivation. Br J Educ Psychol.

[7] Kourmousi N, Darviri C, Varvogli L, Alexopoulos EC. (2015). Teacher Stress Inventory: validation of the Greek version and
perceived stress levels among 3,447 educators. Psychol Res Behav Manag.

[8] Friedman-Krauss AH, Raver CC, Morris PA, Jones SM. (2014). The role of classroom-level child behavior problems in predicting
preschool teacher stress and classroom emotional climate. Early Educ Dev.

[9] Thibaut F. (2017). Anxiety disorders: a review of current literature. Dialogues Clin Neurosci.

[10] Bridgeman PJ, Bridgeman MB, Barone J. (2018). Burnout syndrome among healthcare professionals. Am J Health Syst Pharm.

[11] Weigl M, Schneider A, Hoffmann F, Angerer P. (2015). Work stress, burnout, and perceived quality of care: a
cross-sectional study among hospital pediatricians. Eur J Pediatr.

[12] Silva MCM, Gomes ARS. (2009). Stress ocupacional em profissionais de saúde: um estudo
com médicos e enfermeiros portugueses. Estud Psicol.

[13] Tawfik DS, Profit J, Morgenthaler TI, Satele DV, Sinsky CA, Dyrbye LN (2018). Physician burnout, well-being, and work unit safety grades in
relationship to reported medical errors. Mayo Clin Proc.

[14] Rothenberger DA. (2017). Physician burnout and well-being: A systematic review and
framework for action. Dis Colon Rectum.

[15] Grace MK, VanHeuvelen JS. (2019). Occupational variation in burnout among medical staff: Evidence
for the stress of higher status. Soc Sci Med.

[16] Lipp MN. (2005). Inventário de sintomas de stress para adultos de Lipp.

[17] Jbeili C. (2008). Questionário preliminar de identificação da
burnout.

[18] Kristensen C. (2005). Estresse pós-traumático: sintomatologia e funcionamento
cognitivo [Tese de Doutorado].

[19] Pitthan LO. (2010). Exposição do professor substituto da saúde
ao estresse no trabalho [Dissertação de
Mestrado].

[20] World Health Organization, Calouste Gulbenkian Foundation (2014). Social determinants of mental health.

[21] Chichra A, Abhijnhan A, Tharyan P. (2019). Job stress and satisfaction in faculty of a teaching hospital in
south India: A cross-sectional survey. J Postgrad Med.

[22] Pasqualucci PL, Damaso LLM, Danila AH, Fatori D, Lotufo Neto F, Kochj VHK. (2019). Prevalence and correlates of depression, anxiety, and stress in
medical residents of a Brazilian academic health system. BMC Med Educ.

[23] Collin V, Toon M, O’Selmo E, Reynolds L, Whitehead P. (2019). A survey of stress, burnout and well-being in UK
dentists. Br Dent J.

[24] Souza JA, Fadel CB, Ferracioli MU. (2016). Estresse no cotidiano acadêmico: um estudo com
pós-graduandos em Odontologia. Rev ABENO.

[25] Fisher AT, Alder JG, Avasalu MW. (1998). Lecturing performance appraisal criteria: Staff and student
differences. Aust J Educ.

[26] Rutter H, Herzberg J, Paice E. (2002). Stress in doctors and dentists who teach. Med Educ.

[27] Allen I, Hale RM, Herzberg J, Paice E. (1999). Stress among consultants in North Thames.

[28] Jackson T, Zhou C, Khorgami Z, Jackson D, Agrawal V, Taubman K (2019). Traumatized residents - It’s not surgery. It’s
medicine. J Surg Educ.

[29] Han A, Kim J, Kim J. (2019). Coping strategies, social support, leisure activities, and
physical disabilities. Am J Health Behav.

